# Supplementary material for: The optimization of heterogeneous catalytic conditions in the direct alkylation of waste vegetable oil
Source: R Soc Open Sci. 2020 Jul 22;7(7):192254. doi: 10.1098/rsos.192254 (PMC7428286; doi:10.1098/rsos.192254)
Supplement: The experimental operations and supplentary data [file rsos192254supp1.docx]

## 1 Materials

AlCl_3_, sodium chloroacetate, sodium sulfate anhydrous and N,N-dimethyl-1,3-propanediamine were analytical reagent grade and purchased from the Aladdin Chemical Reagent (Shanghai, China). Benzene, hydrochloric acid, methanol, ethanol and ethyl acetate were purchased from the Shanghai Lingfeng Chemical Reagent Co., Ltd (Shanghai, China).

## 2 Characterization

Gas chromatography mass spectrometry was recorded on Agilent 6890N Network GC system and 5975 inert Mass Selective Detector.

Pyrolysis-gas chromatography/mass spectrometry was recorded on Agilent 7890A-5975C system with pyrolyzer at 600 ^◦^C.

Electrospray ionization mass spectrometry which was recorded on Waters Xevo G2 Tof system.

Determination of the exchange capacity was conducted using back titration.

Elemental analysis was conducted on Elementar Vario EL Ⅲ;

Atomic emission spectrometer was recorded on Inductively Coupled Plasma Atomic Emission Spectrometer;

## 3 Results

### 3.1 The optimum reaction condition

Waste cooking oil, benzene and catalyst were added into a sealed reactor with magnetic stirring at 120-140 ^◦^C for 2-6 h. After completing the reaction, the alkylation product was achieved by filtration.

The conversions were calculated by comparing the change of unsaturated fatty acid methyl esters using methyl stearate as the internal standard substance. The yields were calculated by the peak area (Extracted Molecular Ion Chromatogram) ratio of benzyl methyl stearate to the sum of benzyl methyl stearate and unsaturated fatty acid methyl esters. The selectivity was calculated by the ratio of the yield to conversion.

$$Conversion rate=1-\frac{{{A'}_{unsaturated fatty acid methyl esters}}/{{A'}_{methyl stearate}}}{{A_{unsaturated fatty acid methyl esters}}/{A_{methyl stearate}}}$$

Notes:

A'_unsatuated fatty acid methyl esters_: The peak area of unsaturated fatty acid methyl esters in the GC-MS total ions chromatogram after reactions.

A'_methyl stearate_: The peak area of methyl stearate in the GC-MS total ions chromatogram of after reactions.

A_unsatuated fatty acid methyl esters_: The peak area of unsaturated fatty acid methyl esters in the GC-MS total ions chromatogram of waste vegetable oil.

A_methyl stearate_: The peak area of methyl stearate in the GC-MS total ions chromatogram of waste vegetable oil.

$$Yield=\frac{{A''}_{benzyl methyl stearate}}{{A''}_{unsaturated fatty acid methyl esters}+{A''}_{benzyl methyl stearate}}$$

Notes:

A''_unasturated fatty acid methyl esters_: The peak area of unsaturated acid methyl esters in the GC-MS extracted molecular ion chromatogram after reactions.

A''_benzyl methyl stearate_: The peak area of benzyl methyl stearate in the GC-MS extracted molecular ion chromatogram of after reactions.

$$Selectivity=\frac{Yield}{Conversion rate}$$

Table S1 Orthogonal experiment of the alkylation reaction

| Number | Temperature/^°^C | Solvent ratio(v/v) | Catalyst loading(w/v) | Reaction time/h | Yield |
| --- | --- | --- | --- | --- | --- |
| 1 | 120 | 1 | 0.7 | 2 | 40.11% |
| 2 | 120 | 2 | 1.4 | 4 | 70.14% |
| 3 | 120 | 3 | 2.1 | 6 | 92.53% |
| 4 | 130 | 1 | 1.4 | 6 | 84.64% |
| 5 | 130 | 2 | 2.1 | 2 | 83.44% |
| 6 | 130 | 3 | 0.7 | 4 | 75.85% |
| 7 | 140 | 1 | 2.1 | 4 | 94.90% |
| 8 | 140 | 2 | 0.7 | 6 | 96.57% |
| 9 | 140 | 3 | 1.4 | 2 | 88.41% |
| Average1 | 67.592% | 73.218% | 70.842% | 70.654% |  |
| Average2 | 81.309% | 83.381% | 81.062% | 80.294% |  |
| Average3 | 93.292% | 85.594% | 90.289% | 91.245% |  |
| Range | 25.700% | 12.376% | 19.447% | 20.591% |  |

Notes: Solvent ratio(v/v) = benzene/waste cooking oil ratio

Catalyst loading(w/v) = HND 580/ waste vegetable oil ratio

Table S2 The error analysis of the orthogonal experiment

| Factors | SS | df | F | F_0.1_(2,8) | F_0.05_(2,8) | F_0.01_(2,8) |
| --- | --- | --- | --- | --- | --- | --- |
| Temperature/^°^C | 0.099 | 2 | 1.610 | 3.110 | 4.460 | 8.650 |
| Benzene/oil ratio(v/v) | 0.026 | 2 | 0.423 | 3.110 | 4.460 | 8.650 |
| HND-580/g | 0.057 | 2 | 0.927 | 3.110 | 4.460 | 8.650 |
| Reaction time/h | 0.064 | 2 | 1.041 | 3.110 | 4.460 | 8.650 |
| Error | 0.25 | 8 |  |  |  |  |

As showed in Table S1, the temperature has the greatest effect on the yield. The reaction time and catalyst loading also contributes a lot to the yield while the solvent ratio has the lowest effect on the yield. According to the Arrhenius equation, the reaction rate constant will increase with the rise of temperature, resulting in acceleration of reaction rate and variations in the yields. In addition, the activation energy will be significantly reduced after the introduction of catalyst, and the reaction rate was improved with the increase of catalyst loading. As for the solvent ratio, the introduction of solvent will influence the concentration of the raw materials, resulting in the variations of reaction rate and yields. As for the reaction time, the yields will be improved with the increase of reaction time until the reaction equilibrium is reached

The optimum reaction condition was Temperature = 140 ^°^C, Solvent ratio(*v/v*) =3, catalyst loading(*w/v*) = 0.55, Reaction time =6 h. Furthermore, on the optimum reaction condition, the conversion rate of the waste cooking oil could reach 97.91% in 2 h only.

Thus, Temperature = 140 ^°^C, Solvent ratio(*v/v*) =3, catalyst loading(*w/v*) = 0.55, Reaction time =2 h was the ideal reaction condition where the high conversion rate could be achieved in short time.

### 3.2 Operation life of the catalyst

20 rounds of reactions were done to investigate the operation life of the catalyst. In addition, the crude product collected in every round of reaction was tested by GC-MS and the results were listed in the manuscript as Fig.2

The linear fitting of the experiment data was $\text{y}\text{ }\text{=1.014}\text{ }\text{-}\text{ }\text{0.012}\text{x}$ and the half-life of the catalyst was 42.2 h.

### 3.3 The deactivation mechanism of catalyst

3.3.1 The loss of catalytic center

The metal organic sulfonates in the catalyst were detected by metal content through ICP method and the results were listed in table S3

Table S3 ICP detection of the metallic elements in the catalyst

| Project | Fe^3+^  (mmol/g) | Cr^3+^  (mmol/g) | Ni^2+^  (mmol/g) | Mo^4+^  (mmol/g) | Mn^2+^  (mmol/g) |
| --- | --- | --- | --- | --- | --- |
| The unused catalyst | <1.70*10^-4^ | <3.85*10^-5^ | <3.41*10^-5^ | <2.08*10^-5^ | <3.64*10^-5^ |
| The catalyst after recycle | 0.0304 | 0.00538 | 0.00699 | 0.00103 | 0.000564 |
| The loss of H^+^ | 0.0912 | 0.0161 | 0.0140 | 0.00412 | 0.00113 |
| The loss of exchange capacity | 0.127 mmol/g[H^+^] | | | | |

The contents of metallic elements in used catalyst has grown much higher than before, indicating that corrosion of reactor (made of stainless steel 316) has occurred in the alkylation process. However, the contribution of the corrosion to the declination of the exchange capacity was only 0.127 mmol/g[H^+^] after calculation, about 2.56% of the total exchange capacity. Thus, the corrosion of reactor which caused about 2.56% loss of total exchange capacity was not the key factor causing deactivation of catalyst.

3.3.2 The dysfunction of catalytic center

The exchange capacity determination and elements analysis results and PyGC-MS were listed in table S4, table S5 and Fig.S1, respectively.

Table S4 Exchange capacity of the catalyst

| Project | Exchange capacity (mmol/g[H^+^]) |
| --- | --- |
| The catalyst | 5.00 |
| The catalyst after recycle | 3.82 |

Table S5 Elemental analysis of the catalyst

| Project | C[%] | H[%] | S[%] |
| --- | --- | --- | --- |
| The catalyst | 42.8 | 4.86 | 16.7 |
| The catalyst after recycle | 56.7 | 6.60 | 11.9 |


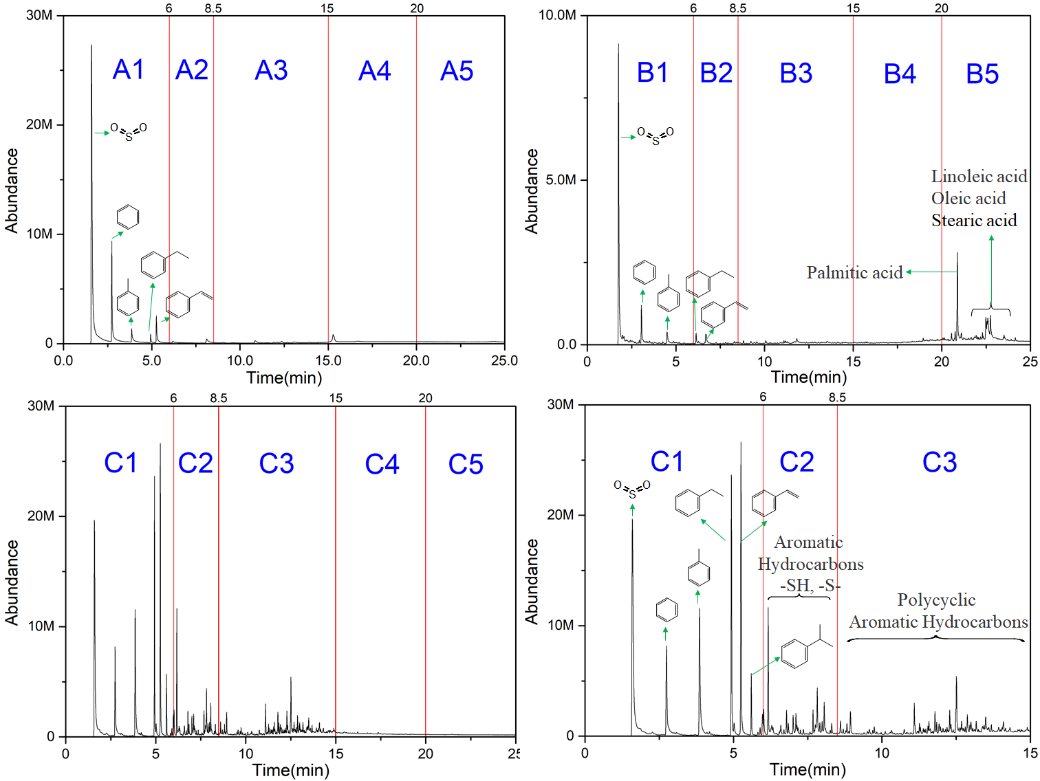


Fig. S1 The PyGC-MS total ion spectra of the catalyst; A: The unused catalyst; B: The catalyst bathed in waste vegetable oil; C: The catalyst after 20 rounds recycle; The spectra were divided into five parts by the categories of the pyrolysis products

### 3.4 The rebirth of the catalyst

3.4.1 Extraction by benzene

The catalyst after 20 rounds recycle was refluxed by 30 mL benzene for 3 h and the oil phase was filtrated and detected by ESI and GC-MS. Then catalyst was treated again in the same way and washed by benzene for five times in total.

In addition, the catalyst dried to constant weight at 105 ^°^C was added into a sealed reactor equipped with magnetic stirring in the optimum condition and the catalytic efficiency tested was 21.9%


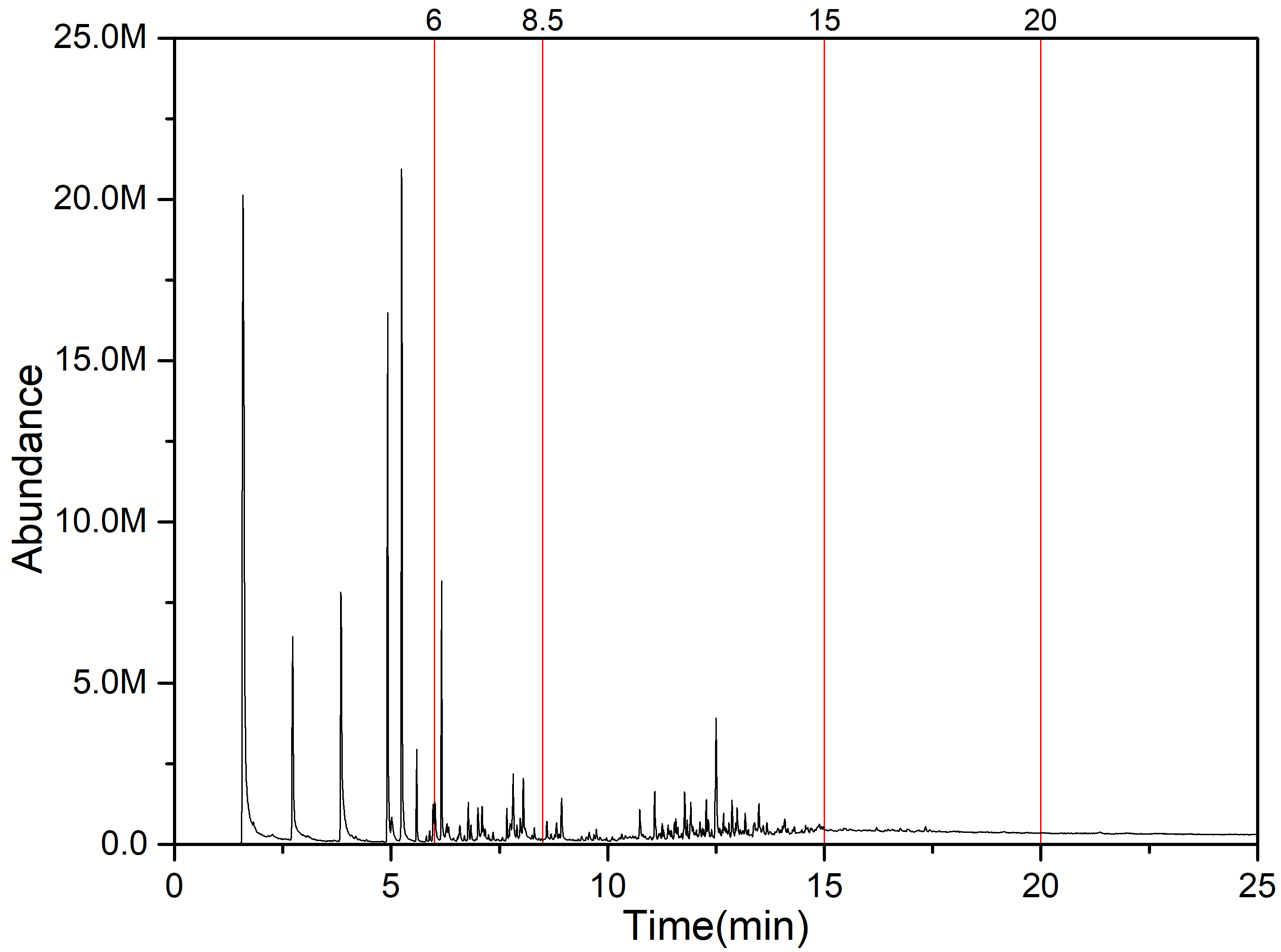


Fig S2 The PyGC-MS total ion spectra of the rebirthed catalyst (Extraction by benzene)

3.4.2 Saponification by alkali

The catalyst after 20 rounds recycle was saponificated by 30 mL 0.1mol/L NaOH solutions and the water phase was filtrated and detected by ESI and GC-MS. Then catalyst was treated again in the same way and saponificated by 30 mL 0.1mol/L NaOH solutions for three times in total.

After saponification, H type catalyst was transformed into Na type catalyst which could be rebirthed into H type catalyst again by ion exchange with dilute acid. The rebirthed catalyst dried to constant weight at 105 ^°^C was added into a sealed reactor equipped with magnetic stirring in the optimum condition and the catalytic efficiency tested was 55.9%.


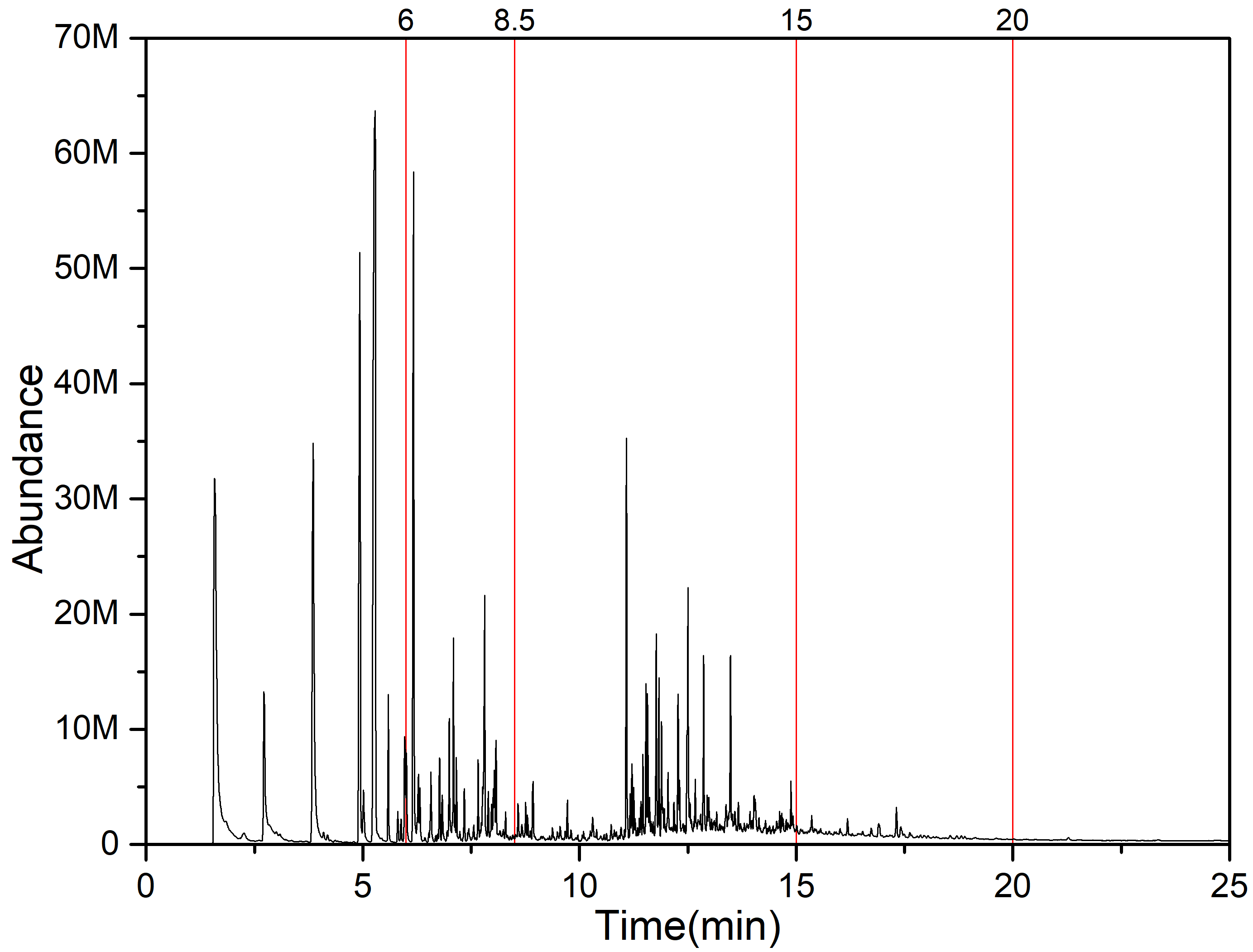


Fig. S3 The PyGC-MS total ion spectra of the rebirthed catalyst (Saponification by alkali)

### 3.5 The two-step alkylation of waste cooking oil

5 mL fatty acids ethyl esters from waste cooking oil, 15 mL benzene and 11 g HND 580 were added into a sealed reactor equipped with magnetic stirring at 140 ^°^C for 14 h. After completing the reaction, the reactor was first cooled to the room temperature and then the crude product was collected by filtration while the catalyst was used in the next round of reaction. The crude product collected in every round of reaction was tested by GC-MS and the results were listed in Fig. 4


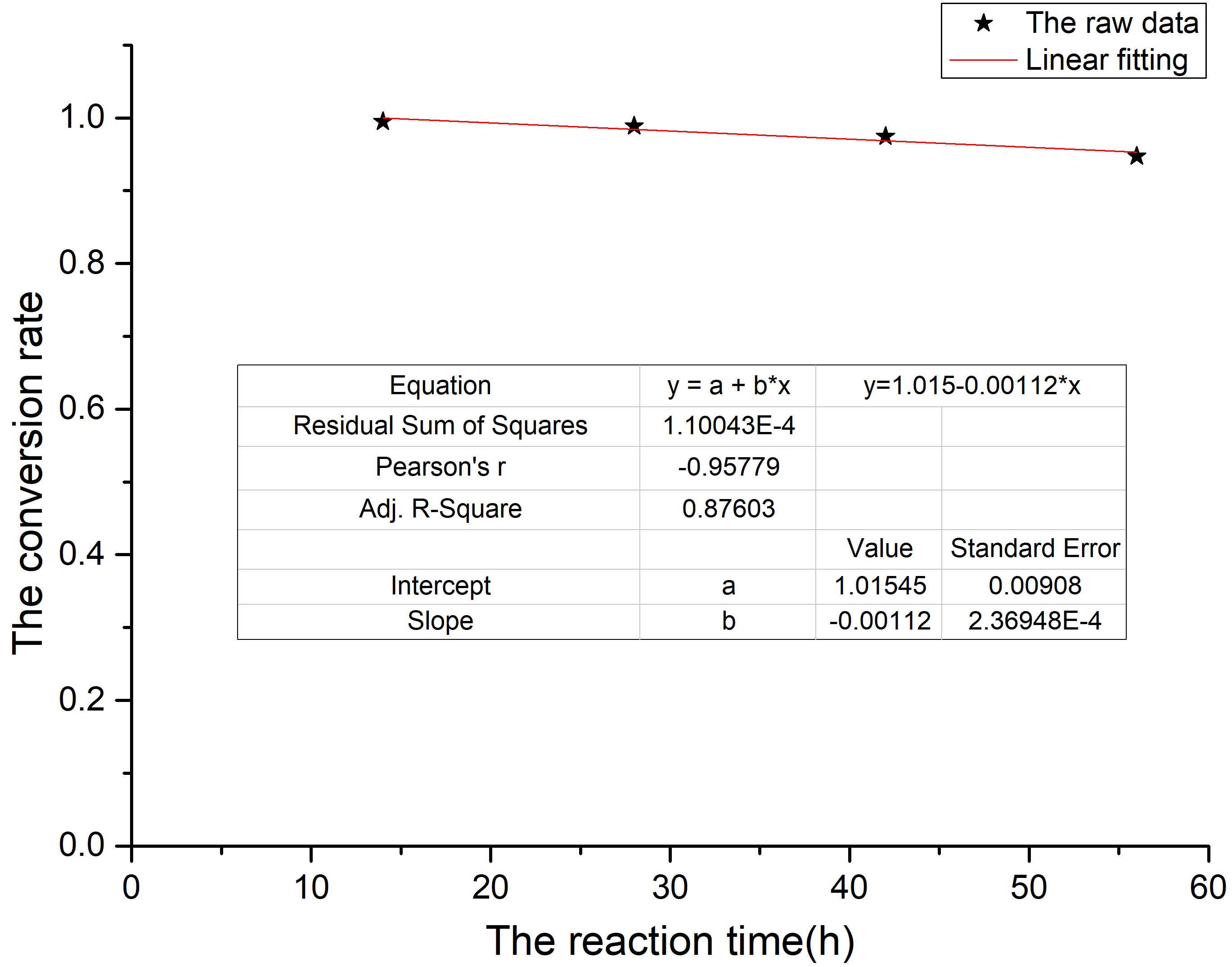


Fig. S4 The effect of the recycle rounds on the catalytic efficiency

The curve fitting equation of the raw data was $\text{y}\text{ }\text{=1.015}\text{ }\text{-}\text{ }\text{0.0}\text{01}\text{12}\text{x}$ and the half-life of the catalyst was 453 h.

After washed by benzene for three times and dried to constant weight at 105 ^°^C, the catalyst after 56 h reaction was detected by GC-MS and the conversion rate was 95.9%

### 3.6 The detection of the catalyst before and after uses

3.6.1 Specific surface area

As for five solid catalysts, the surface area acquired from the product specification was all above 20 m^2^/g. The specific area of solid catalyst HND580 before and after uses was detected and the results were listed in Table S6

Table S6 The specific surface area of the solid catalyst

| Project | BET Surface Area | Langmuir Surface Area |
| --- | --- | --- |
| The catalyst before uses | 19.17 m^2^/g | 26.15 m^2^/g |
| The catalyst after uses | 24.43 m^2^/g | 36.52 m^2^/g |

As showed in Table S6, the specific surface area of solid catalyst has increased after uses. The insoluble polymers blocked in the solid catalyst could decrease the specific surface area of the solid catalyst. However, the damage and wearing of solid catalyst may increase the specific surface area of the solid catalyst. Thus, it hard to confirm the deactivation mechanism only by the detection of specific surface area.

3.6.3 Fourier Transform infrared spectroscopy (FTIR)

The catalyst before and after uses were tested by FTIR and the results were listed as follows.


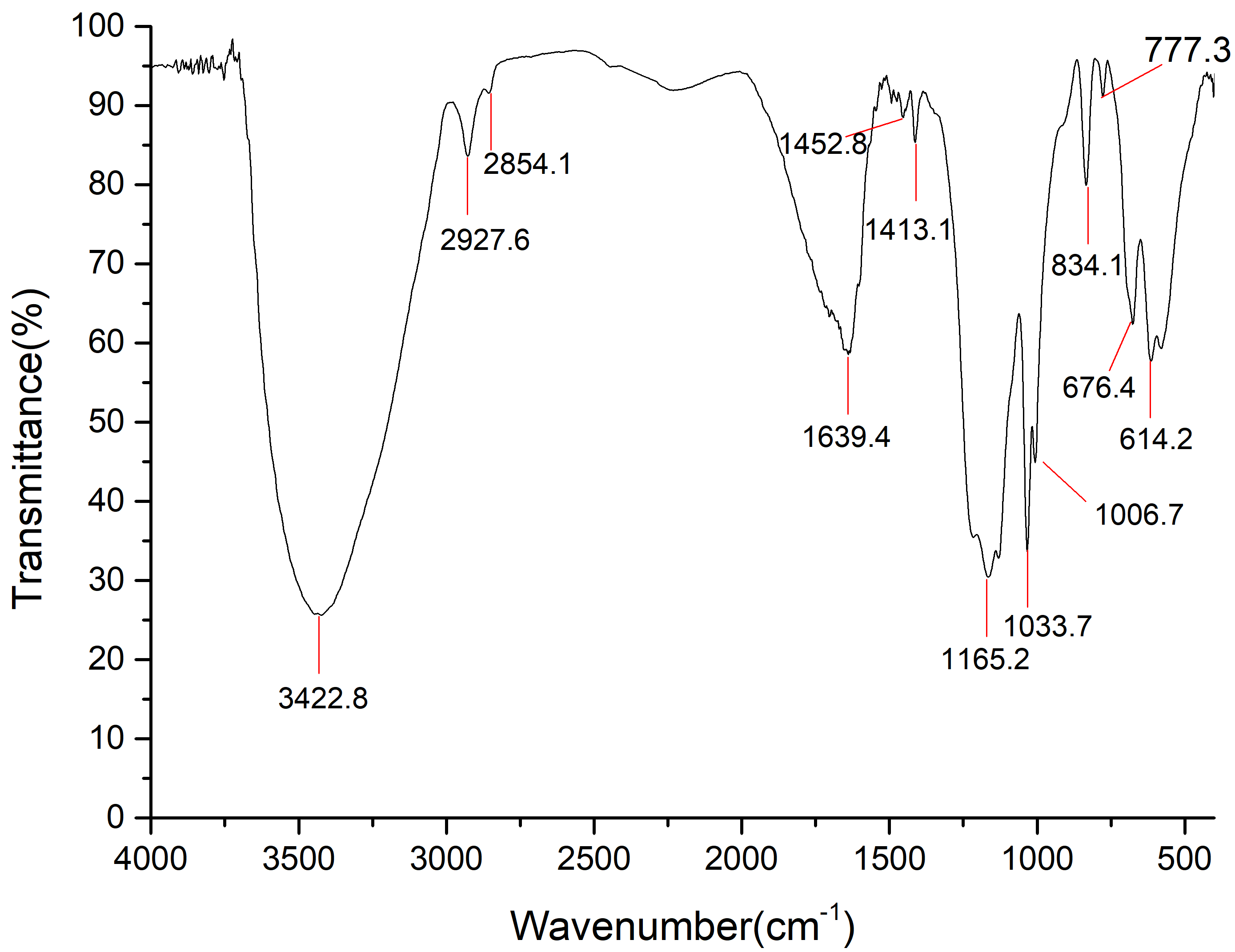


Fig.S5 Fourier Transform infrared spectroscopy of the solid catalyst before uses


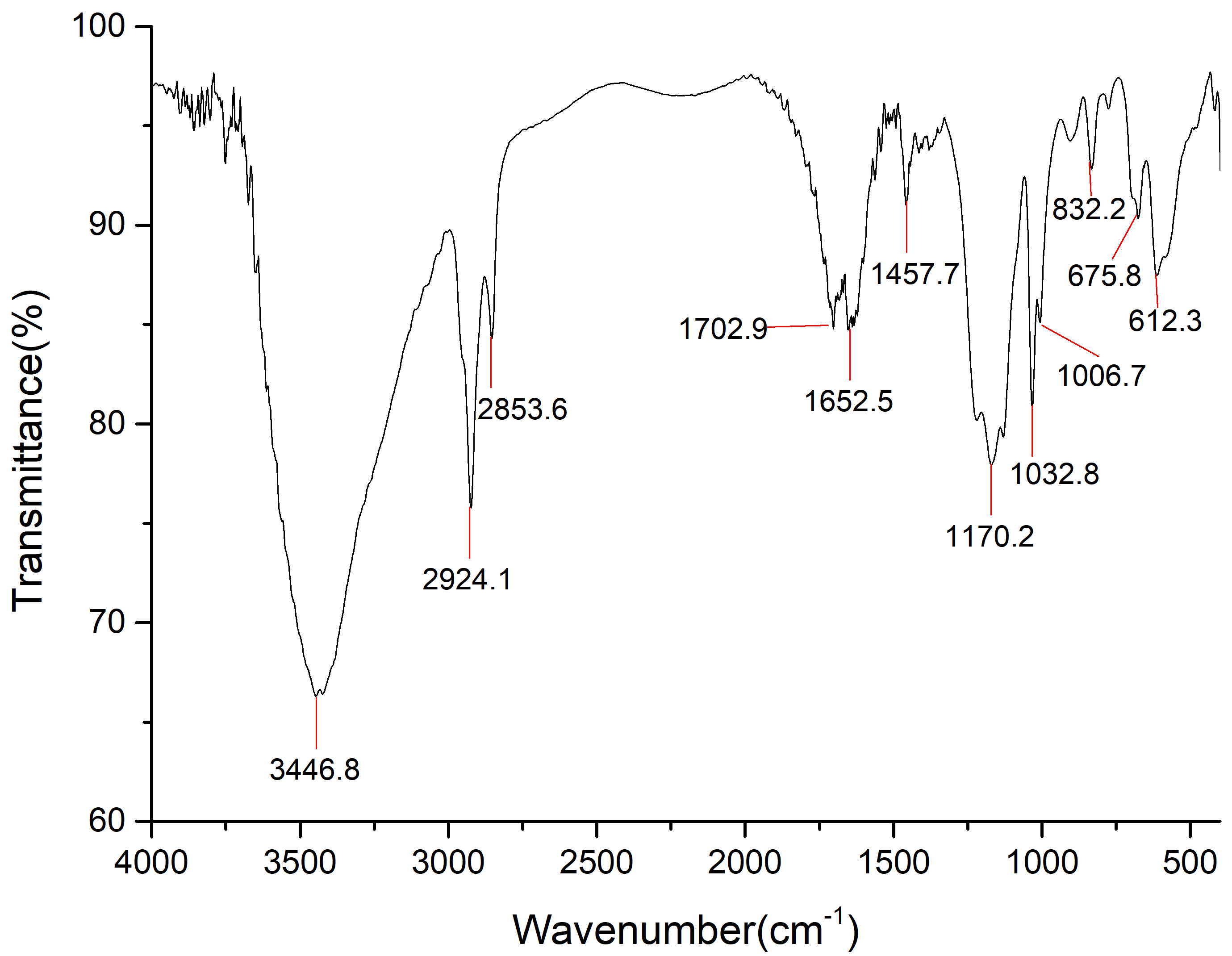


Fig.S6 Fourier Transform infrared spectroscopy of the solid catalyst after uses

Catalyst before uses: FTIR: 3422.8 cm^-1^(H_2_O), 2927.6 cm^-1^(-CH_2_-, *v*_asCH_), 2854.1 cm^-1^(-CH2-, *v*_sCH_), 1639.4 cm^-1^ /1450 cm^-1^ (benzene, C=C), 834.1 cm^-1^/777.3 cm^-1^/676.4 cm^-1^(benzene, =C-H), 1165.2 cm^-1^(-SO_2_-OH).

Catalyst after uses: FTIR: 3446.8 cm^-1^(H_2_O), 2924.1 cm^-1^(-CH_2_-, *v*_asCH_), 2853.6 cm^-1^(-CH2-, *v*_sCH_), 1702.9 cm^-1^(C=O), 1652.5 cm^-1^ /1457 cm^-1^ (benzene, C=C), 832.2 cm^-1^/675.8 cm^-1^ (benzene, =C-H), 1170.2 cm^-1^(-SO_2_-OH).

As for -CH_2_-, the transmittance had been greatly increased for the catalyst after uses. In addition, the absorption of C=O indicated that new substances were attached to the catalyst after uses. Above all, some fatty acid derivatives might be attached to the catalyst.

3.6.4 X-ray Photoelectron Spectroscopy(XPS)

The distribution of elements was tested by XPS and the results were listed as follows.

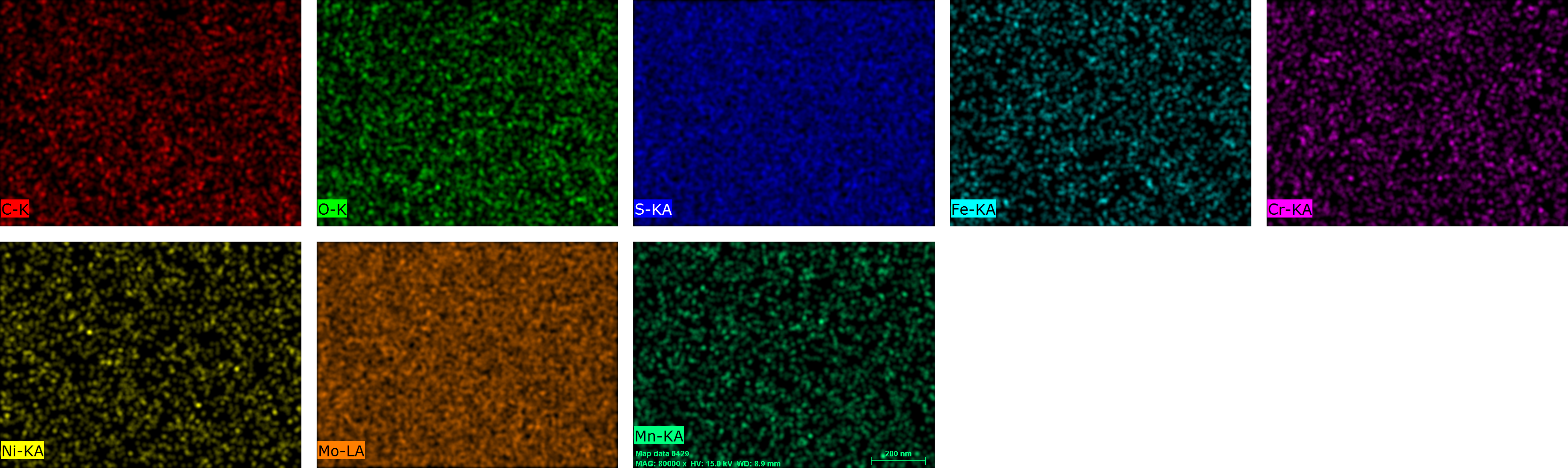


Fig.S7 XPS(C/O/S/Fe/Cr/Ni/Mo/Mn) of the solid catalyst before uses


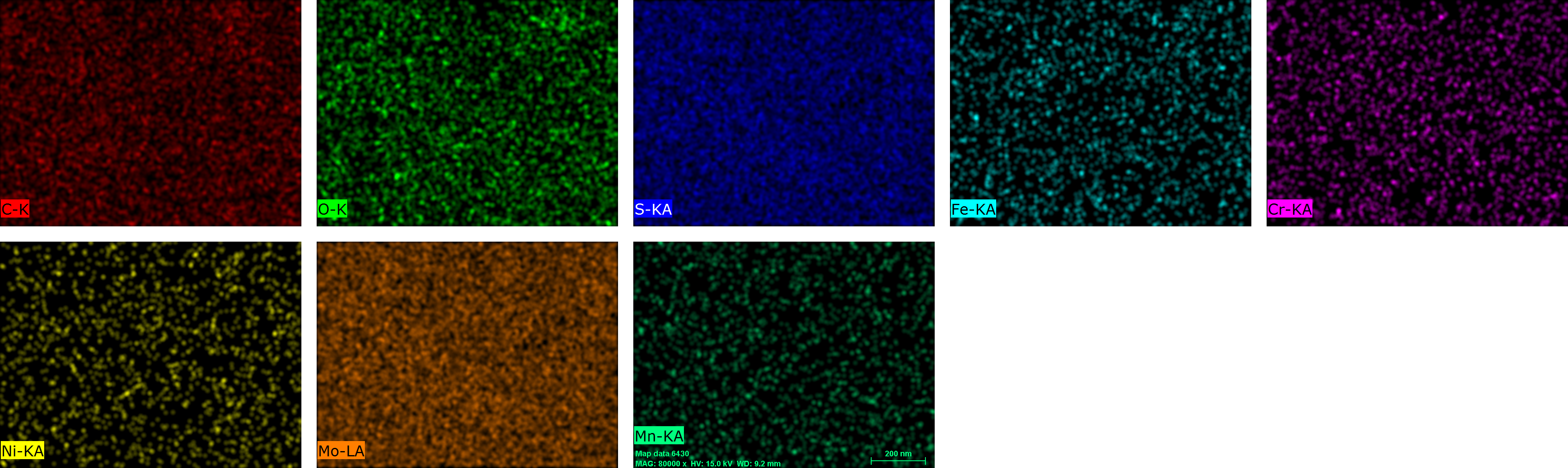


Fig.S8 XPS(C/O/S/Fe/Cr/Ni/Mo/Mn) of the solid catalyst after uses

It could be inferred from the XPS results that there were C/O/S/Fe/Cr/Ni/Mo/Mn elements in the solid catalyst. Moreover, the peak of sulfur element has not changed significantly, indicating that the catalytic center has not lost greatly and the loss of catalytic center may not be the major factor causing the catalyst deactivation.
